# New or little-known *Boreoheptagyia* (Diptera, Chironomidae) in China inferred from morphology and DNA barcodes

**DOI:** 10.3897/zookeys.1040.66527

**Published:** 2021-05-28

**Authors:** Xiao-Long Lin, Hai-Jun Yu, Xin-Hua Wang, Wen-Jun Bu, Chun-Cai Yan, Wen-Bin Liu

**Affiliations:** 1 College of Life Sciences, Nankai University, Tianjin, 300071, China Nankai University Tianjin China; 2 Center of Animal husbandry and Fisheries, Bijiang Agriculture and Rural affairs Bureau, Tongren, Guizhou, 554300, China Center of Animal husbandry and Fisheries, Bijiang Agriculture and Rural affairs Bureau Guizhou China; 3 Tianjin Key Laboratory of Conservation and Utilization of Animal Diversity, Tianjin Normal University, Tianjin, 300387, China Tianjin Normal University Tianjin China

**Keywords:** COI, Diamesinae, integrative taxonomy, new species, non-biting midges

## Abstract

The male adult of *Boreoheptagyia zhengi* Lin & Liu, **sp. nov.** is described and illustrated based on material collected in China. Associated morphological characteristics and reference to its DNA barcode are provided. *Boreoheptagyia kurobebrevis* (Sasa & Okazawa, 1992) is newly recorded from China based on both a male and female, with additional associated data on the DNA barcode of the male adult. A neighbor-joining tree based on available *Boreoheptagyia* DNA barcodes and a key to the adults of *Boreoheptagyia* from China are given.

## Introduction

Brundin (1966) erected the genus *Boreoheptagyia* with *Heptagyia rugosa* Saunders, 1930 as type species by original designation. Larval populations of this genus live in cool, fast-flowing streams and other harsh environments (Thienemann 1954; Oliver 1989). At present, the genus includes 24 valid species worldwide (Ashe and O’Connor 2009). Among them, 17 are restricted to the Palaearctic Region including the two recently described species *B. joeli* Makarchenko, 2020 and *B. ortladamellica* Rossaro, 2017 (Rossaro 2017; Makarchenko et al. 2020), seven to the Oriental Region and one to the Nearctic Region. Only *B. rotunda* Serra-Tosio, 1983 occurs in both Palaearctic and Oriental regions (Ashe and O’Connor 2009; Rossaro 2017). Seven species are currently reported from China: *B. alulasetosa* Makarchenko, Wu & Wang, 2008, *B. ambigua* Makarchenko, Wu & Wang, 2008, *B. brevitarsis* (Tokunaga, 1936), *B. similis* (Chaudhuri & Ghosh, 1981), *B. tibetica* Makarchenko, Wang & Willassen, 1996, *B. xinglongiensis* Makarchenko, Wu & Wang, 2008 and *B. joeli* Makarchenko, 2020 (Makarchenko et al. 1996; Wang 2000; Makarchenko et al. 2008; Makarchenko et al. 2020).

The DNA barcode corresponding to the 658-bp fragment of the mitochondrial gene cytochrome *c* oxidase I (COI) has been identified as the core of a global bio-identification system at the species level (Hebert et al. 2003a, b) and has proved to be useful in the delimitation of non-biting midge species and has provided important evidence to confirm new species (Anderson et al. 2013; Lin et al. 2015; Giłka et al. 2018; Lin et al. 2018; Song et al. 2018; Lin et al. 2019; Liu et al. 2021).

In the present study, morphology and the DNA barcode of *B. zhengi* Lin & Liu, sp. nov. are provided based on material collected in Yunnan Province, China. *Boreoheptagyia kurobebrevis* (Sasa & Okazawa, 1992) is newly recorded from China based on a male and female, the latter was associated with the male by standard DNA barcodes. DNA barcode analysis including the partial COI sequences of species of genus *Boreoheptagyia* is conducted. A key to the known adults of *Boreoheptagyia* from China is also given.

## Materials and methods

The examined adults were preserved in 85% ethanol and stored in the dark at 4 °C before morphological and molecular analyses. Genomic DNA was extracted from the thorax and head using a Qiagen DNA Blood and Tissue Kit at Nankai University, Tianjin, China (**NKU**), following the standard protocol (Lin et al. 2018) except for the final elution volume of 100 µl. After DNA extraction, the exoskeleton of each specimen was mounted in Euparal on a microscope slide together with the corresponding wings, legs, antennae and abdomen, following the procedures outlined by Sæther (1969). Morphological terminology follows Sæther (1980).

Digital photographs of the mounted specimens were taken at 300-dpi resolution using a Nikon Digital Sight DS-Fil camera mounted on Nikon Eclipse 80i compound microscope using the software NIS-Elements F v.4.60.00. at the College of Life Sciences, Nankai University, Tianjin, China (NKU).

The universal primers LCO1490 and HCO2198 (Folmer et al. 1994) were used to amplify the standard 658-bp mitochondrial COI barcode region. Polymerase chain reaction (PCR) amplifications followed Song et al. (2018) and were conducted in a 25 μl volume including 12.5 μl 2× Es Taq MasterMix (CoWin Biotech Co., Beijing, China), 0.625 μl of each primer, 2 μl of template DNA and 9.25 μl of deionized H_2_O. PCR products were electrophoresed in 1.0% agarose gel, and purified and sequenced in both directions at Beijing Genomics Institute Co., Ltd., Beijing, China.

Raw sequences were assembled and edited in Geneious Prime 2020 (Biomatters Ltd., Auckland, New Zealand). Alignment of the sequences was carried out using the MUSCLE algorithm (Edgar 2004) on amino acids in MEGA X (Kumar et al. 2018). The pairwise distances using the Kimura 2-Parameter (K2P) substitution model of ten species within the genus *Boreoheptagyia* were calculated in MEGA. The neighbor-joining tree was constructed using the K2P substitution model, 1000 bootstrap replicates and the “pairwise deletion” option for missing data in MEGA. Novel sequence, trace-files, and metadata of the new species are uploaded to the Barcode of Life Data Systems (BOLD) (Ratnasingham and Hebert 2013). GenBank accessions of the Chinese specimens are list in Table 1. The holotype of the new species and other examined specimens are deposited in the collection of the College of Life Sciences, Nankai University, Tianjin, China.

**Table 1.** Kimura 2-parameter pairwise genetic distances based on COI barcodes of the *Boreoheptagyia*.

Species	Pairwise genetic distances																						GenBank accessions
*Boreoheptagyia zhengi*																							MZ128909
*Boreoheptagyia brevitarsis*	15.1																						MZ128906
*Boreoheptagyia kurobebrevis*	14.9	14.1																					MZ128908
	14.9	14.1	0.0																				MZ128907
*Boreoheptagyia joeli*	13.7	11.2	12.9	12.9																			MT240752
	14.3	11.9	13.5	13.5	0.9																		MT240753
	14.1	11.9	13.3	13.3	0.8	1.1																	MT240754
	13.9	11.8	13.1	13.1	0.8	0.5	0.9																MT240755
*Boreoheptagyia sarymsactyensis*	13.9	13.6	15.9	15.9	11.0	10.8	11.1	10.6															MT240756
	14.1	13.8	16.1	16.1	11.2	11.0	11.3	10.8	0.2														MT240757
	14.3	13.4	16.1	16.1	10.8	10.6	11.0	10.4	0.8	0.6													MT240758
*Boreoheptagyia brevitarsis*	12.1	11.8	12.9	12.9	9.2	9.9	9.9	9.7	11.7	11.9	12.2												MT240774
*Boreoheptagyia* sp. EAM-2017	13.2	12.5	13.3	13.3	10.3	10.1	10.4	9.9	7.0	7.2	7.5	11.0											KY640386
*Boreoheptagyia alulasetosa*	17.1	15.7	16.6	16.6	12.7	13.3	13.1	12.9	14.2	14.4	14.4	14.9	15.1										MZ128904
*Boreoheptagyia iranica*	15.6	14.9	14.4	14.4	13.3	13.7	14.2	13.7	16.1	16.3	16.3	13.8	14.0	15.7									MT240768
	15.6	14.9	14.4	14.4	13.3	13.7	14.2	13.7	16.1	16.3	16.3	13.8	14.0	15.7	0.0								MT240769
	15.6	14.9	14.4	14.4	13.3	13.7	14.2	13.7	16.1	16.3	16.3	13.8	14.0	15.7	0.0	0.0							MT240770
	15.6	14.9	14.4	14.4	13.3	13.7	14.2	13.7	16.1	16.3	16.3	13.8	14.0	15.7	0.0	0.0	0.0						MT240771
*Boreoheptagyia brevitarsis*	12.5	12.7	13.9	13.9	10.4	11.1	11.1	11.0	12.4	12.6	12.9	1.2	11.7	14.7	14.2	14.2	14.2	14.2					MT240772
	11.6	12.3	13.7	13.7	9.6	10.1	10.2	9.9	11.5	11.7	12.0	0.8	11.0	14.9	14.0	14.0	14.0	14.0	1.5				MT240773
	14.7	0.5	13.9	13.9	11.0	11.7	11.9	11.5	13.3	13.5	13.1	11.7	12.6	15.7	15.0	15.0	15.0	15.0	12.6	12.2			MZ128905
	11.8	11.9	13.1	13.1	9.6	10.1	10.2	9.9	11.8	12.0	12.4	0.5	10.8	15.3	13.6	13.6	13.6	13.6	1.4	0.8	11.9		MT240775
	11.7	12.3	13.5	13.5	9.9	10.2	10.2	10.1	11.7	11.8	12.2	0.8	11.1	14.9	14.2	14.2	14.2	14.2	1.9	0.9	12.2	1.1	MT240775

## Results

### Taxonomic description

#### Boreoheptagyia kurobebrevis

Taxon classificationAnimaliaDipteraChironomidae

(Sasa & Okazawa, 1992)

F1AF6E57-6EE3-5E79-A6AC-42471FE04595

Figs 1 , 2 , 3 , 4

Diamesa kurobebrevis Sasa & Okazawa, 1992: 58.
Toyamadiamesa kurobebrevis Sasa & Kikuchi, 1995: 205.
Boreoheptagyia kurobebrevis Endo, 2002: 12; Makarchenko et al. 2008: 8; Ashe and O’Connor 2009: 265.

##### Material examined.

Male (NKU & BOLD sample ID: LGS63), 1 male, China, Guizhou Province, Qiandongnan Miao and Dong Autonomous Prefecture, Leishan, Leigongshan Natural Reserve, Fangxiang, Getoucun, 26.396014°N, 108.260933°E, 1070 m a.s.l., Malaise trap, 10–30. i. 2020, leg. H.-J. Yu. 1 female, same as above.

##### Diagnostic characters.

*B. kurobebrevis* can be distinguished from other related species in having: antenna with five flagellomeres; wing membrane covered with microtrichia on entire surface. Costal extension 175 μm long. R with 31 setae, R_1_ with 34 setae, R_4+5_ with 31 setae. Superior volsella rounded; inferior volsella finger-like and well-sclerotized; gonostylus with 2 small megasetae.

##### Adult male

(*N* = 1). Total length 2.95 mm. Wing length 2.60 mm. Total length/wing length 1.13. Wing length/length of profemur 2.17.

**Figure 1.**
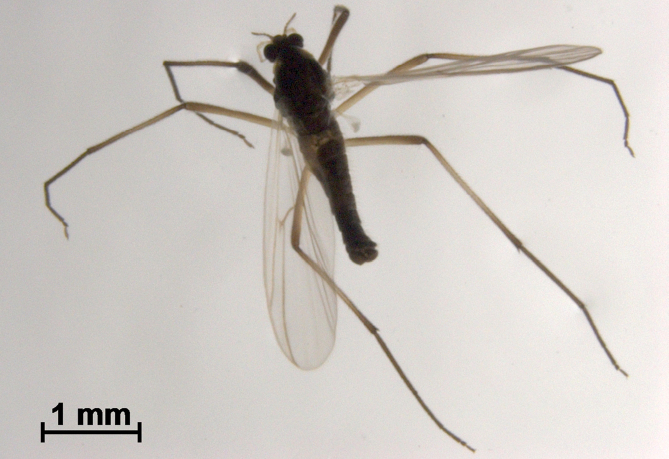
*Boreoheptagyia kurobebrevis* (Sasa & Okazawa, 1992), male adult in ethanol.

***Coloration*** (Fig. 1). Head, thorax and abdomen mostly dark brown. Ground color of scutum yellow, stripes and postnotum dark brown, scutellum brown, abdominal tergites almost uniformly yellow, hypopygium dark brown, femur yellow in basal three-quarter with brown ring apically, tibia pale medially with brown rings in basal one-fifth and distal one-third.

***Head*.** Antenna with five flagellomeres. AR 0.82. Temporal setae 8, not separable into inner and outer verticals. Clypeus with 20 setae. Tentorium 75 µm long; 25 µm wide. Lengths of palpomere 1–5 (in µm): 38, 50, 105, 158, 250. Length ratio of palpomeres 5/3: 2.38.

**Figure 2.**
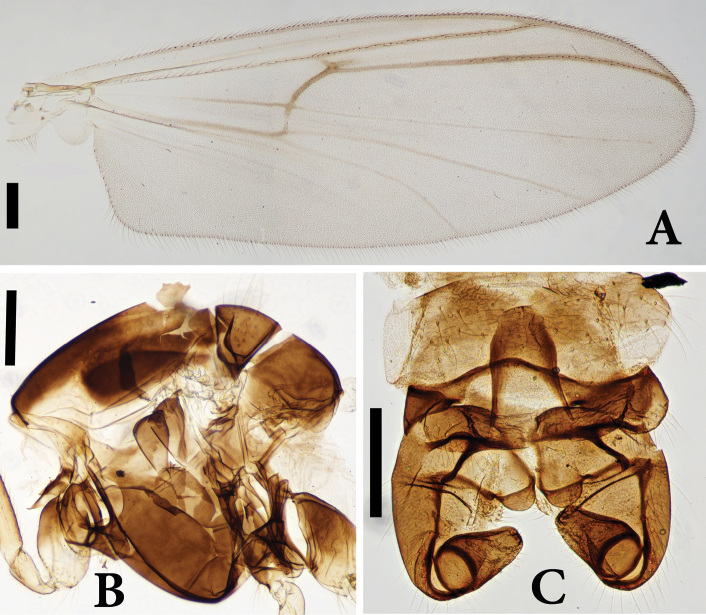
*Boreoheptagyia kurobebrevis* (Sasa & Okazawa, 1992), male adult **A** wing **B** thorax **C** hypopygium. Scale bars: 200 µm.

***Thorax*** (Fig. 2B). Antepronotum with 9 anterolateral setae; acrostichals 28; dorsocentrals 17 in two rows; prealars 10. Scutellum with 46 setae.

***Wing*** (Fig. 2A). Brachiolum with 5 setae; wing membrane with macrotrichia on entire surface; costal extension 175 µm long. Distribution of setae on veins: R, 31; R_1_, 34; R_4+5_ 3. Anal lobe well-developed; squama with 16 setae. VR 1.08.

***Legs*.** Length (in µm) of spurs of: P_1_, 40; P_2_, 55 and 55; P_3_, 80 and 50. Width (in µm) of tibial apex of: P_1_, 60; P_2_, 70 µm; P_3_, 100. Comb on hind tibia with Comb of hind tibia with 12 setae. Lengths (in µm) and proportions of legs as in Table 2.

**Table 2.** Lengths (in µm) and proportions of legs of *Boreoheptagyia kurobebrevis* (Sasa & Okazawa, 1992) in China, male (*N* = 1).

	fe	ti	ta_1_	ta_2_	ta_3_	ta_4_	ta_5_	LR	BV	SV	BR
P_1_	1200	1380	920	500	270	80	130	0.67	3.57	2.80	2.64
P_2_	1300	1220	750	480	240	70	120	0.61	3.59	3.36	3.50
P_3_	1260	1450	900	500	250	70	90	0.62	3.97	3.01	3.89

***Hypopygium*** (Fig. 2C). Tergites IX without anal point. Tergites IX with 30 setae.

***Gonocoxite*** 300 µm long. Superior volsella rounded, 60 µm long. Inferior volsella well-sclerotized, finger-like, bearing 17 setae. Gonostylus 140 µm long, with two small megasetae. HR 2.14; HV 2.11.

##### Genitalia of female

**Figure 3.**
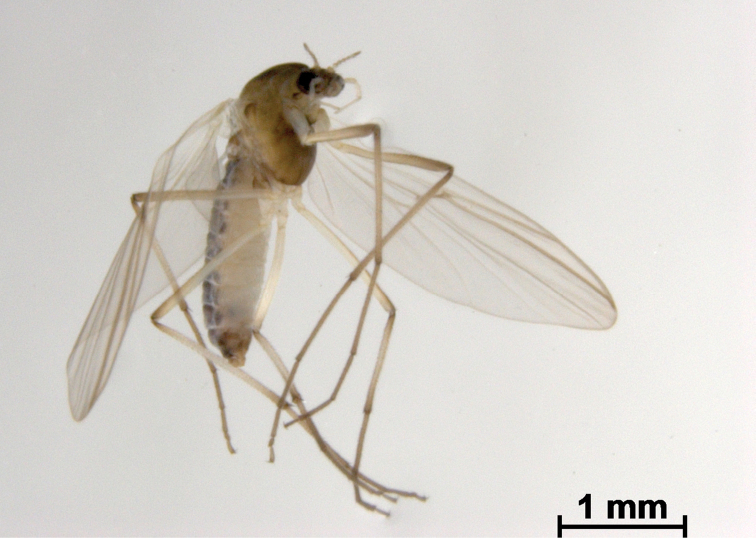
*Boreoheptagyia kurobebrevis* (Sasa & Okazawa, 1992), female adult.

**Figure 4.**
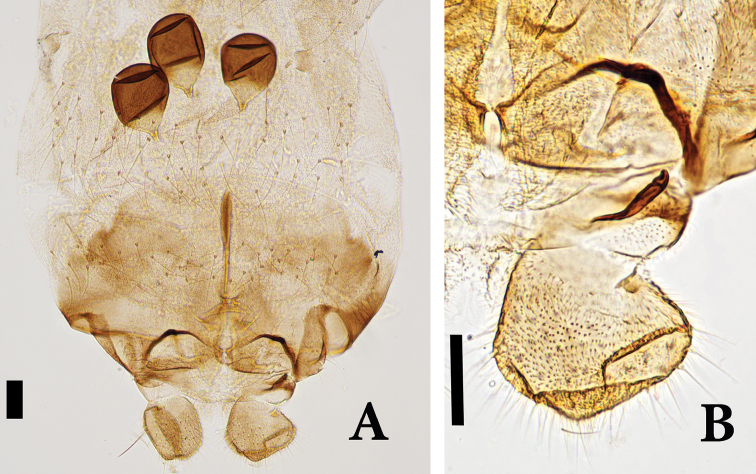
*Boreoheptagyia kurobebrevis* (Sasa & Okazawa, 1992), female adult **A** genitalia with gonapophysis VIII and seminal capsules, ventral **B** cercus. Scale bars: 50 µm.

(*N* = 1) (Figs 3, 4). *Gonocoxite* IX 100 µm long, 45 µm wide, with 7 setae. Cercus 88 μm long, 75 μm wide, with 47 setae and covered with macrotrichia. Seminal capsule 158 μm long, 75 μm wide, sclerotized part 98 μm long.

#### Boreoheptagyia zhengi

Taxon classificationAnimaliaDipteraChironomidae

Lin & Liu sp. nov.

9CB72FA8-D8D5-5CA2-BF72-37D5FE47D54F

http://zoobank.org/9B5BB346-3355-469A-9F0E-125C350041E7

Figs 5 , 6

##### Type material.

***Holotype*:** male (NKU & BOLD sample ID: XL3519), Yunnan Province, Baoshan City, Mangkuan County, Gaoligongshan National Nature Reserve, Baihualing, near a remote wild hot spring and a stream, 25.3105556°N, 98.795000°E, 1475 m a.s.l., light trap, 22.v.2018, leg. X.-L. Lin.

##### Diagnostic characters.

According to the morphological characters of the adult male, the new species keys to the genus *Boreoheptagyia*. The new species is distinguished from its other congeners by the following combination of characters: antenna with seven flagellomeres; wing membrane covered with microtrichiae on almost entire surface except a bare area near anal lobe; femora and tibiae of all legs pale in basal half, other portions brown; superior volsella tongue shape with small projection; gonostylus with one megaseta.

##### Adult male

(*N* = 1). Total length 2.10 mm. Wing length 1.42 mm. Total length/wing length 1.48. Wing length/length of profemur 1.63.

**Figure 5.**
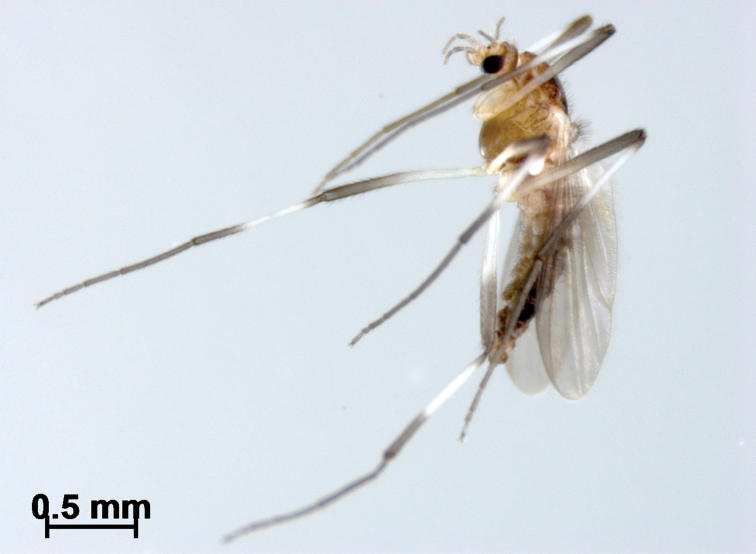
*Boreoheptagyia zhengi* Lin & Liu, sp. nov., male adult, holotype.

***Coloration*** (Fig. 5). Head, thorax and abdomen yellow-brown. Wing membrane with microtrichiae on almost entire surface except a bare area near anal lobe. Femur and tibia of all legs pale in basal half, other portions brown.

**Figure 6.**
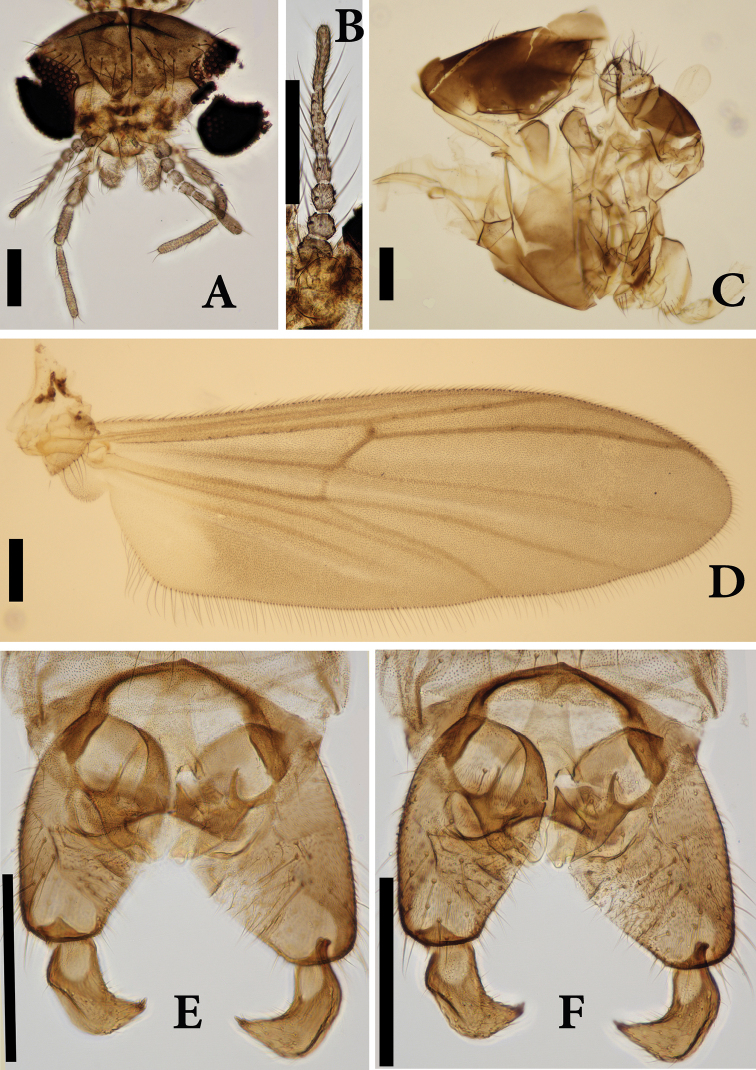
*Boreoheptagyia zhengi* Lin & Liu, sp. nov., male adult, holotype **A** hea **B** antenna **C** thorax **D** wing **E** hypopygium, dorsal view **F** hypopygium, ventral view. Scale bars: 100 µm.

***Head*** (Fig. 6A, B). Antenna with seven flagellomeres; ultimate flagellomere 55 µm long; AR 0.31. Eyes bare without dorsomedial extension. Temporal setae 14, not separable into inner and outer verticals. Clypeus with nine setae. Lengths of palpomere 1–5 (in µm): 20, 30, 60, 80, 150. Palpomere ratio (5^th^/3^rd^): 2.50.

***Thorax*** (Fig. 6C). Antepronotum with five setae; acrostichals 27; dorsocentrals 14 in two rows; prealars five. Scutellum with 30 setae.

***Wing*** (Fig. 6D). VR 1.04. Brachiolum with three setae. Costa without extension. Squama with eight setae. R with 11 setae, R_1_ with four setae, R_4+5_ with five setae. Anal lobe developed.

***Legs*.** Spur of front tibia 35 µm long, of mid tibia 38 µm long; of hind tibia 55 and 33 µm long. Width of front tibia apex 50 µm, of mid tibia apex 50 µm, of hind tibia apex 63 µm. Comb of hind tibia with 15 setae. Lengths (in µm) and proportions of legs as in Table 3.

**Table 3.** Lengths (in µm) and proportions of legs of *Boreoheptagyia zhengi* Lin & Liu, sp. nov., male holotype (*N* = 1).

	fe	ti	ta_1_	ta_2_	ta_3_	ta_4_	ta_5_	LR	BV	SV	BR
P_1_	870	810	440	200	120	50	80	0.54	4.71	3.82	2.57
P_2_	860	850	480	220	120	50	100	0.56	4.47	3.56	3.91
P_3_	920	890	510	250	120	50	100	0.57	4.46	3.55	4.23

***Hypopygium*** (Fig. 6E, F). Tergites IX without anal point. Tergites IX with 23 setae. Gonocoxite 200 µm long. Superior volsella tongue shape with small projection, 50 µm long. Inferior volsella sclerotized, finger-like, 30 µm long. Gonostylus 75 µm long, with one megaseta, 10 µm long. HR 2.67; HV 2.80.

Female and immature stages unknown.

##### Etymology.

The species is named ‘*zhengi*’ after Prof. Le-Yi Zheng, for his outstanding contribution to the knowledge of insect taxonomy in China; noun in nominative case.

### Key to the known adult males of *Boreoheptagyia* Brundin from China

**Table:** 

1	Antenna with 13 flagellomeres	**2**
–	Antenna less than 9 flagellomeres	**6**
2	Alula with 3–4 setae. Gonostylus short and inflated, with very short, narrow apical part	***B. alulasetosa* Makarchenko, Wu & Wang**
–	Alula without setae. Shape of gonostylus different	**3**
3	Dorsocentrals only in single anterior group on scutum	**4**
–	Dorsocentrals in anterior and posterior groups on scutum	**5**
4	Prealars 12. Inferior volsella with some distal teeth	***B. ambigua* Makarchenko, Wu & Wang**
–	Prealars 1–4. Inferior volsella without teeth	***B. xinglongiensis* Makarchenko, Wu & Wang**
5	Prealars 17–28	***B. brevitarsis* (Tokunaga)**
–	Prealars 10	***B. similis* (Chaudhuri & Ghosh)**
6	Antenna with 8–9 flagellomeres	***B. joeli* Makarchenko**
–	Antenna with 6–7 flagellomeres	**7**
7	Wing developed, antenna with 7 flagellomeres	***B. zhengi* Lin & Liu, sp. nov.**
–	Wing reduced, antenna with 6 flagellomeres	***B. tibetica* Makarchenko, Wang & Willassen**

## Discussion

Morphological characters of *B. kurobebrevis* from China fit well with the original description by Sasa and Okazawa (1992) and Makarchenko et al. (2008), but there are a few differences in numeric measurements: total length (2.95 mm), AR 0.82 and scutellum with 46 setae in Chinese specimen, as compared with total length 3.34 mm, AR 0.64 and scutellum with 50 setae in Japanese specimen (Makarchenko et al. 2008).

The new species can be easily separated from other related members of the genus by the following combination of morphological characters found in the male adult: antenna with seven flagellomeres; wing membrane covered with macrotrichia on almost the entire surface except a bare spot near the anal lobe. *Boreoheptagyia zhengi* sp. nov. keys out close to *B. tibetica* from which it can be separated in having: 1) antenna with seven flagellomeres in *B. zhengi* Lin & Liu, sp. nov., whereas the latter has six flagellomeres; 2) well-developed anal lobe in the new species and the wing membrane with microtrichiae on almost the entire surface except for a bare area near the anal lobe, whereas *B. tibetica* has a reduced anal lobe and wing membrane with macrotrichia on the entire surface; 3) differing number of chaetae on thorax: (acrostichals 27, dorsocentrals 14 in two rows, prealars 5 in the new species) compared with (acrostichals 14, dorsocentrals 6, prealars 15–16 in *B. tibetica*).

**Figure 7.**
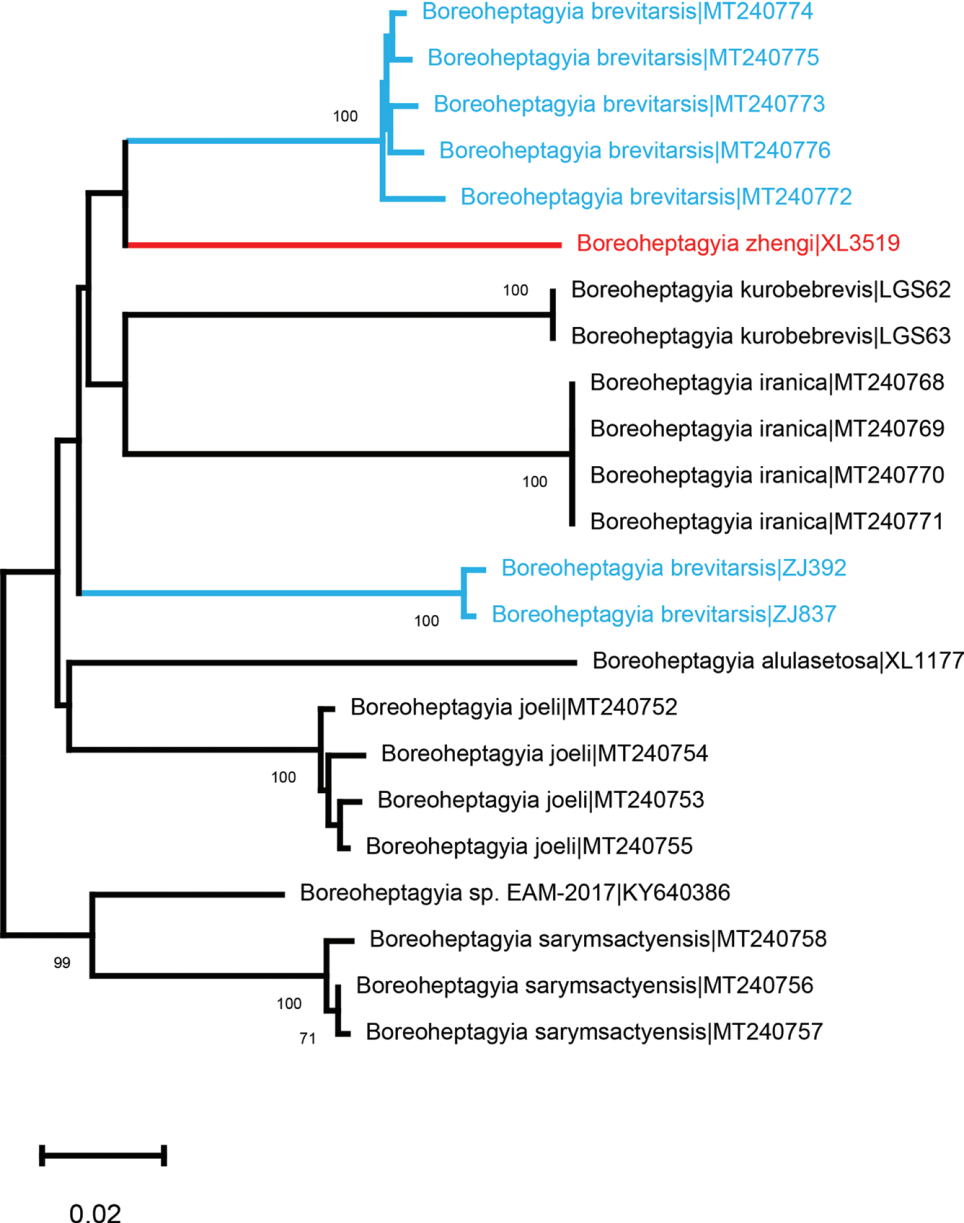
Neighbor-joining tree for six species of the genus *Boreoheptagyia* based on K2P distance in DNA barcodes. Numbers on branches represent bootstrap support (>70%) based on 1000 replicates; scale equals K2P genetic distance.

The neighbor-joining tree based on COI DNA barcodes of *Boreoheptagyia* revealed nine distinct genetic clades (Fig. 7). The new species *B. zhengi* sp. nov. separates from *B. brevitarsis* by more than 11% divergence in COI barcodes (Fig. 7; Table 1). In addition, there are two genetic clades of *Boreoheptagyia brevitarsis* (Fig. 7), indicating that a potential cryptic species or misidentification. A further integrative taxonomic study on *Boreoheptagyia brevitarsis* is needed when the more public vouchers are available to access.

## Supplementary Material

XML Treatment for Boreoheptagyia kurobebrevis

XML Treatment for Boreoheptagyia zhengi

## References

[B1] Anderson AM Stur E Ekrem T (2013) Molecular and morphological methods reveal cryptic diversity and three new species of Nearctic *Micropsectra* (Diptera: Chironomidae). Freshwater Science 32: 892–921. 10.1899/12-026.1

[B2] Ashe P O’Connor JP (2009) A World Catalogue of Chironomidae (Diptera). Part 1. Buchonomyiinae, Chilenomyiinae, Podonominae, Aphroteniinae, Tanypodinae, Usambaromyiinae, Diamesinae, Prodiamesinae and Telmatogetoninae.– Irish Biogeographical Society and National Museum of Ireland, Dublin, 445 pp.

[B3] Brundin L (1966) Transantarctic relationships and their significance, as evidenced by chironomid midges. With a monograph of the subfamilies Podonominae and Aphroteniinae and the austral Heptagyiae. Kungliga Svenska Vetensk Apsakademiens Handlingar 11(1): 1–472.

[B4] Edgar RC (2004) MUSCLE: multiple sequence alignment with high accuracy and high throughput. Nucleic Acids Research 32: 1792–1797. 10.1093/nar/gkh340 15034147 PMC390337

[B5] Endo K (2002) List of Boreoheptagyia from Japan. Yusurika 23: 1–12. [In Japanese]

[B6] Folmer O Black M Hoeh W Lutz R Vrijenhoek R (1994) DNA primers for amplification of mitochondrial cytochrome *c* oxidase subunit I from diverse metazoan invertebrates. Molecular Marine Biology and Biotechnology 3: 294–299. 7881515

[B7] Giłka W Paasivirta L Gadawski P Grabowski M (2018) Morphology and molecules say: *Tanytarsus latens*, sp. nov. from Finland (Diptera: Chironomidae). Zootaxa 4471: 569–579. 10.11646/zootaxa.4471.3.8 30313397

[B8] Hebert PDN Cywinska A Ball SL (2003a) Biological identifications through DNA barcodes. Proceedings of the Royal Society of London B: Biological Sciences 270: 313–321. 10.1098/rspb.2002.2218 PMC1691236 12614582

[B9] Hebert PDN Ratnasingham S de Waard JR (2003b) Barcoding animal life: cytochrome *c* oxidase subunit 1 divergences among closely related species. Proceedings of the Royal Society of London B: Biological Sciences 270: S96–S99. 10.1098/rsbl.2003.0025 PMC1698023 12952648

[B10] Kumar S Stecher G Li M Knyaz C Tamura K (2018) MEGA X: molecular evolutionary genetics analysis across computing platforms. Molecular Biology and Evolution 35(6): 1547–1549. 10.1093/molbev/msy096 29722887 PMC5967553

[B11] Lin XL Stur E Ekrem T (2015) Exploring genetic divergence in a species-rich insect genus using 2790 DNA Barcodes. PLoS ONE 10: e0138993. 10.1371/journal.pone.0138993 PMC4583400 26406595

[B12] Lin XL Stur E Ekrem T (2018) DNA barcodes and morphology reveal unrecognized species of Chironomidae (Diptera). Insect Systematics & Evolution 49: 329–398. 10.1163/1876312X-00002172

[B13] Lin XL Yu HJ Zhang RL Wang XH (2019) Polypedilum (Cerobregma) heberti sp. n. (Diptera: Chironomidae) from Gaoligong Mountains, Yunnan, China. Zootaxa 4571(2): 255–262. 10.11646/zootaxa.4571.2.5 31715818

[B14] Liu WB Yao Y Yan CC Wang XH Lin XL (2021) A new species of *Polypedilum* (Cerobregma) (Diptera, Chironomidae) from Oriental China. ZooKeys 1011: 139–148. 10.3897/zookeys.1011.59554 33568962 PMC7847467

[B15] Makarchenko EA Wang X Willassen E (1996) A new species of *Boreoheptagyia* Brundin (Diptera, Chironomidae) from Tibet (China). Japanese Journal of Entomology 64: 825–829.

[B16] Makarchenko EA Endo K Wu J Wang X (2008) A review of *Boreoheptagyia* Brundin, 1966 (Chironomidae: Diamesinae) from East Asia and bordering territories, with the description of five new species. Zootaxa 1817: 1–17. 10.11646/zootaxa.1817.1.1

[B17] Makarchenko E Semenchenko E Palatov D (2020) Taxonomy of some Boreoheptagyiini Brundin (Diptera: Chironomidae: Diamesinae) from the mountains of Central Asia and the Middle East, with description and DNA barcoding of new taxa. Zootaxa 4790(1): 91–107. 10.11646/zootaxa.4790.1.5 33055856

[B18] Oliver DR (1989) The adult males of Diamesinae (Diptera:Chironomidae) of the Holarctic region – Keys and diagnoses In: Wiederholm T (Ed.) Chironomidae of the Holarctic region. Keys and diagnoses. Part 3 – Adult males.Entomologica Scandinavica Supplement 34: 129–154.

[B19] Ratnasingham S Hebert PDN (2013) A DNA-based registry for all animal species: the barcode index number (BIN) system. PLoS ONE 8: e66213. 10.1371/journal.pone.0066213 PMC3704603 23861743

[B20] Rossaro B (2017) *Boreoheptagyia ortladamellica* sp. nov. (Diptera, Chironomidae) from Italian Alps. Journal of Entomological and Acarological Research 49: 77–80. 10.4081/jear.2017.6860

[B21] Sæther OA (1969) Some Nearctic Podonominae, Diamesinae, and Orthocladiinae (Diptera: Chironomidae). Bulletin of the Fisheries Research Board of Canada 170: 1–154.

[B22] Sæther OA (1980) Glossary of chironomid morphology terminology (Diptera: Chironomidae). Entomologica Scandinavica Supplement 14: 1–51.

[B23] Sasa M Okazawa T (1992) Studies on the chironomid midges (yusurika) of Kurobe River. Research Report from Toyama Prefectural Environmental Pollution Research Centre 40–91.

[B24] Sasa M Kikuchi M (1995) Chironomidae (Diptera) of Japan. University of Tokyo Press, Tokyo, 333 pp.

[B25] Song C Lin XL Wang Q Wang XH (2018) DNA barcodes successfully delimit morphospecies in a superdiverse insect genus. Zoologica Scripta 47: 311–324. 10.1111/zsc.12284

[B26] Thienemann A (1954) *Chironomus*. leben, Verbreitung und wirtschaftliche Bedeutung der Chironomiden. Binnengewässer 20: 1–834.

[B27] Wang XH (2000) A revised checklist of chironomids from China (Diptera). In: Hoffrichter O (Ed.) Late 20th Century Research on Chironomidae: an Anthology from the 13th International Symposium on Chironomidae.Shaker Verlag, Achen, 629–652.

